# 28-day sepsis mortality prediction model from combined serial interleukin-6, lactate, and procalcitonin measurements: a retrospective cohort study

**DOI:** 10.1007/s10096-022-04517-1

**Published:** 2022-11-16

**Authors:** Yinjing Xie, Dehua Zhuang, Huaisheng Chen, Shiqing Zou, Weibu Chen, Yue Chen

**Affiliations:** 1 Department of Medical Laboratory, Shenzhen People’s Hospital, The Second Clinical Medical College of Jinan University, The First Affiliated Hospital of South University of Science and Technology, Shenzhen, 518020 Guangdong China; 2 Department of Critical Care Medicine, Shenzhen People’s Hospital, The Second Clinical Medical College of Jinan University, The First Affiliated Hospital of South University of Science and Technology, Shenzhen, 518020 Guangdong China

**Keywords:** Prediction model, Sepsis, Mortality, Interleukin-6, Lactate, Procalcitonin

## Abstract

Sepsis is a global medical issue owing to its unacceptably high mortality rate. Therefore, an effective approach to predicting patient outcomes is critically needed. We aimed to search for a novel 28-day sepsis mortality prediction model based on serial interleukin-6 (IL-6), lactate (LAC), and procalcitonin (PCT) measurements. We enrolled 367 septic patients based on Sepsis-3 (Third International Consensus Definitions for Sepsis and Septic Shock). Serum IL-6, LAC, and PCT levels were measured serially. Results collected within 24 and 48–72 h of admission were marked as D1 and D3 (e.g., IL-6D1/D3), respectively; the IL-6, LAC, and PCT clearance (IL-6c, LACc, PCTc) at D3 were calculated. Data were split into training and validation cohorts (7:3). Logistic regression analyses were used to select variables to develop models and choose the best one according to the Akaike information criterion (AIC). Receiver operating characteristic curves (ROC), calibration plots, and decision curve analysis (DCA) were used to test model performance. A nomogram was used to validate the model. There were 314 (85.56%) survivors and 53 (14.44%) non-survivors. Logistic regression analyses showed that IL-6D1, IL-6D3, PCTD1, PCTD3, and LACcD3 could be used to develop the best prediction model. The areas under the curves (AUC) of the training (0.849, 95% *CI*: 0.787–0.911) and validation cohorts (0.828, 95% *CI*: 0.727–0.929), calibration plot, and the DCA showed that the model performed well. Thus, the predictive value of the risk nomogram was verified. Combining IL-6D1, IL-6D3, PCTD1, PCTD3, and LACcD3 may create an accurate prediction model for 28-day sepsis mortality. Multiple-center research with a larger quantity of data is necessary to determine its clinical utility.

## Introduction

Sepsis has become a major health issue worldwide owing to its high mortality rate [1]. It occurs when an inadequate host response to infection causes life-threatening organ dysfunction [2]. Conquering sepsis was ranked as one of the highest-priority tasks by the World Health Organization (WHO) during the 70th World Health Assembly in 2017, and governments worldwide have been urged to invest greater efforts in achieving this goal [3]. However, it remains a challenge for clinicians to identify high-risk patients with sepsis. Early mortality prediction is of great importance for timely and intensive management, which is a high priority for improving the outcomes of sepsis [4–6]. Traditional prediction models rely mostly on scoring systems that require many variables that are difficult to obtain [7, 8]. Otherwise, the majority of existing models have shortcomings, including tedious processes and poor operability [9]. There is still a lack of effective, simple, and convenient models for predicting the prognosis of sepsis [10]. However, several biomarkers have been shown to play an important role in predicting patient outcomes, we develop a 28-day sepsis mortality prediction model, according to the transparent reporting of a multivariable prediction model for individual prognosis or diagnosis (TRIPOD) [11].

Cytokine storms have been one of the hottest research topics in sepsis [12]. Among these, major pro-inflammatory cytokines, interleukin-6 (IL-6) reflects sepsis at the acute stage [13]. Several studies have assessed the diagnostic and prognostic value of IL-6 in patients [13–16]. However, its prognostic value for predicting sepsis outcomes remains controversial. Some studies concluded that IL-6 levels had a high value in predicting 28-day sepsis mortality [17, 18]; on the other hand, IL-6 was not associated with survival in sepsis [15]. In this study, IL-6 levels were measured consecutively for 72 h from admission, and IL-6 clearance was calculated on D3 (48–72 h) to assess the prognostic value of IL-6 in sepsis.

Lactate (LAC) is an important biomarker of cellular metabolism and energy production [19]. Elevated LAC levels indicate tissue hypoxia. It has been demonstrated as a valuable prognostic marker for hypoperfusion, especially in sepsis, and as a predictor of 28-day sepsis mortality [16]. Some studies have reported that LAC levels and clearance are both predictors of mortality in sepsis [20–22]. Nevertheless, studies on the prognostic value of LAC levels and clearance for sepsis have been limited since the release of the Sepsis-3 criteria [20].

PCT is a protein produced by the thyroid gland that is undetectable in healthy individuals [23]. Since PCT was first described as an infection marker in 1993, it has been reported as a potential marker for assessing the presence of infection and clearance of inflammation and has been used as a guide for antibiotic therapy and prediction of mortality [24]. In the past two decades, it has become the most popular and widely studied sepsis biomarker [14]. Moreover, some studies have suggested that PCT clearance may serve as an outcome predictor in sepsis [25, 26]. However, the performance characteristics of PCT are still insufficient to make it the gold standard for sepsis survival [24]. Thus, our study aimed to evaluate the combined predictive performance of PCT (including PCT levels and clearance) as well as of IL-6 and LAC.

The present study was established to preliminarily develop a novel simple prediction model for the prognosis of 28-day mortality of sepsis by combining the predictive performance of the levels and clearance of IL-6, LAC, and PCT.

## Materials and methods

### Study design and setting

We conducted this retrospective study at the Emergency Department of Shenzhen People’s Hospital. There were 3105 beds in the hospital and 120 beds in the emergency department (ER). An average of approximately 200 patients visit the ER daily. A total of 367 patients diagnosed with Sepsis-3 were included in our study from September 2019 to December 2021. This study was approved by the Ethics Committee of Shenzhen People’s Hospital (no. KY-LL–2,020,157–02). Informed consent was secured from all the participants.

### Study population

We consecutively enrolled adult patients examined and assessed by a physician to have sepsis according to the Sepsis-3 guidelines at the ER. The following were excluded: (1) patients < 18 years of age, (2) patients resuscitated from cardiopulmonary arrest, (3) those with incomplete data requested during the first 48 h of hospitalization (i.e., one measurement), (4) patients on do-not-resuscitate status, (5) patients hospitalized for < 24 h, (6) patients with cancer, and (7) patients who underwent major surgeries in the previous 30 days. Patients with cancer were excluded, since tumors are proposed to use lactate as a fuel, which expands the metabolic functions in cancer [27]. Furthermore, PCT levels will rise in cancer patients. On the other hand, those who underwent major surgeries were excluded because studies have reported elevated IL-6 levels after surgery and returned to baseline levels 2 weeks post-operation [28].

### Data collection

The following clinical data were obtained: age, sex, comorbidities, mechanical ventilation (MV) settings, mean arterial blood pressure (MAP), vasopressor use, type and source of infection, and days of hospital and ICU stay. The outcome was all-cause mortality at 28 days. The worst SOFA score was seen in each patient within 24 h of admission. We measured IL-6 and LAC levels using the Cobas 6000 analyzer (Roche Diagnostics System, Rotkreuz, Switzerland) and PCT levels using the VIDAS immunoassay system (bioMérieux, Marcy L’Etoile, France). All tests were completed during hospitalization in the emergency ward. Results collected within 24 and 48–72 h after admission were marked as D1 and D3, respectively (IL-6D1/D3, LACD1/D3, and PCTD1/D3). All data were retrospectively collected without any intervention. The IL-6, LAC, and PCT clearance values were calculated using the following formula (taking PCT as an example) [20, 29]:

$$\mathrm{PCTc}\;\mathrm D3=\frac{\mathrm{initial}\;\mathrm{PCT}-\mathrm{PCT}\;\mathrm D3}{\mathrm{initial}\;\mathrm{PCT}}$$

### Statistical evaluation

If continuous variables had a normal distribution, they were presented as means ± standard deviations or as medians with interquartile ranges (IQRs). The chi-squared test or Fisher’s exact test was used to compare categorical variables, and the independent two-sample test or Mann–Whitney *U* test was used to compare continuous variables. Missing data were filled with multiple imputations using the MICE package in the R software (version 3.4.3). Then, the data were split into a training cohort and a validation cohort at a ratio of 7:3. Single factor linear regression was used to screen independent variables related to dependent variables (*P* < 0.05); multiple stepwise regression (forward–backward method, both) was performed on the selected independent variables to screen independent influencing factors related to dependent variables, and the AIC was used to determine the optimal model. ROC curves, calibration plots, and DCA were used to test the performance of the model. A nomogram was used to validate the model.

## Results

### Patient demographics

**Fig. 1:**
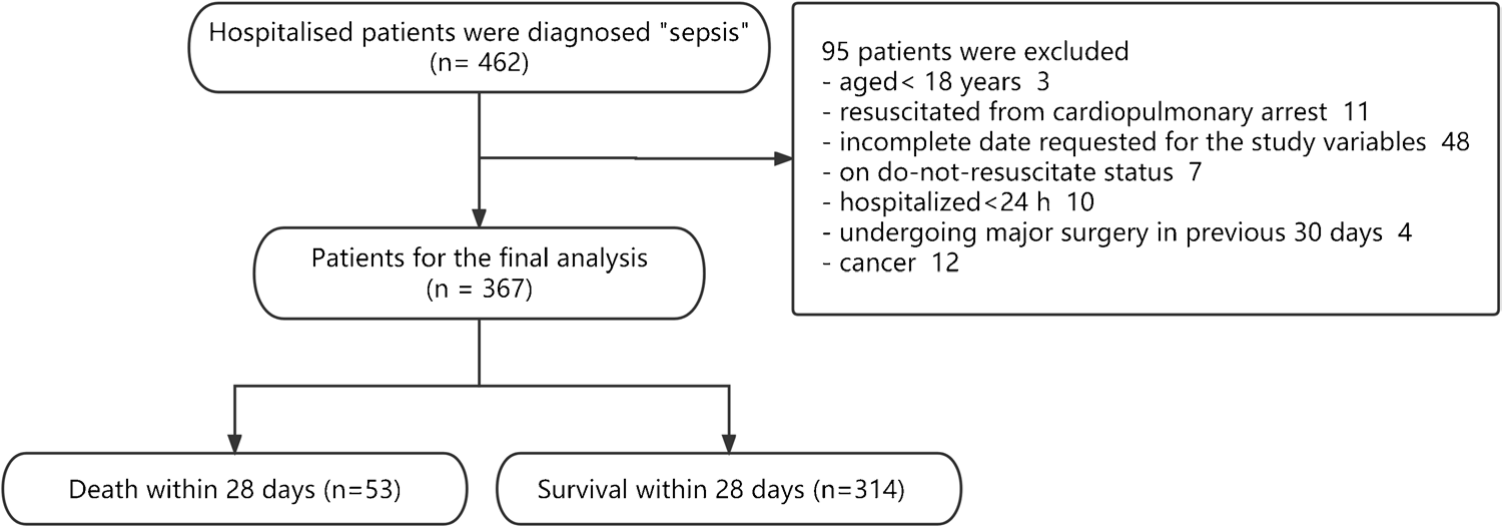
Flow chart of the study population

A total of 462 patients with sepsis were admitted to the ER. We enrolled 367 patients in this study (Fig. 1). After 28 days, there were 314 survivors and 53 mortalities, causing a mortality rate of 14.44%. Table 1 shows the patient demographics. The median (range) patient age was 73 (19–98) years, and 65.9% were male. The most common comorbidity was hypertension (47.4%). The causes of sepsis were pneumonia (68.9%), urinary tract infection (31.1%), intra-abdominal infection (22.6%), and hepatobiliary infection (14.4%). Microbial culture would be conducted twice for each site of the primary infection. Furthermore, 34.9% of the patients had two causes of infection; 9.0% patients had three causes of infection;, and 1.4% had four causes of infection. The pathogens of the infections were gram-positive bacteria (25.3%), gram-negative bacteria (8.2%), and fungi (12.5%).

**Table 1:** Demographic characteristics

Characteristics	Survivor group (*n* = 314)	Non-survivor group (*n* = 53)	Total (*n* = 367)	*P* value
Age, median (range)	71 (19–98)	80 (46–97)	73 (19–98)	0.001*
Sex, male, *n* (%)	215 (65.3)	37 (69.8)	252 (65.9)	0.537
Comorbidities				
Hemiplegic stroke, *n* (%)	12 (3.8)	9 (17.0)	21 (5.7)	0.000*
Diabetes mellitus, *n* (%)	119 (37.9)	17 (32.1)	136 (37.1)	0.417
Hypertension, *n* (%)	146 (46.5)	28 (52.8)	174 (47.4)	0.393
Cardiac insufficiency, *n* (%)	41 (13.1)	5 (9.4)	46 (12.5)	0.461
Chronic renal failure, *n* (%)	61 (19.4)	18 (34.0)	79 (21.5)	0.017*
Coronary heart disease, *n* (%)	27 (8.6)	11 (20.8)	48 (10.4)	0.007*
Coagulation disorder, *n* (%)	41 (13.1)	14 (26.4)	55 (15.0)	0.012*
VTE, *n* (%)	4 (1.3)	0	4 (1.1)	0.912
COPD, *n* (%)	44 (14.0)	16 (30.2)	60 (16.3)	0.003*
Site of primary infection				
Pneumonia, *n* (%)	213 (67.8)	40 (75.5)	253 (68.9)	0.266
Hepatobiliary infection, *n* (%)	43 (13.7)	10 (18.9)	53 (14.4)	0.322
Urinary tract infection, *n* (%)	100 (31.8)	14 (26.4)	114 (31.1)	0.429
Intra-abdominal infection, *n* (%)	72 (22.9)	11 (20.8)	83 (22.6)	0.726
Soft tissue infection, *n* (%)	3 (1.0)	1 (1.9)	4 (1.1)	1.000
Skin and musculoskeletal infections, *n* (%)	6 (1.9)	1 (1.9)	7 (1.9)	1.000
Blood-borne infection, *n* (%)	15 (4.8)	2 (3.8)	17 (4.6)	1.000
Other infection, *n* (%)	6 (1.9)	1 (1.9)	7 (1.9)	1.000
Isolated organisms				
Gram negative bacteria, *n* (%)	73 (23.2)	20 (37.7)	93 (25.3)	0.025*
Gram positive bacteria, *n* (%)	23 (7.3)	7 (13.2)	30 (8.2)	0.240
Fungi, *n* (%)	33 (10.5)	13 (24.5)	46 (12.5)	0.004*
SOFA, mean (SD)	5.6 (2.1)	8.8 (3.4)	7.2 (3.9)	0.001*
ICU stay (day), median (range)	4 (0–45)	7 (0–60)	4 (0–60)	0.001*

*COPD* chronic obstructive pulmonary diseases, *VTE* venous thromboembolism

^*^Indicates a significant value, *P* < 0.05

### Comparison of IL-6, LAC, and PCT levels and clearance between survivor and non-survivor groups

Table 2 provides a comparison of the levels and clearance of IL-6, LAC, and PCT between survivors and non-survivors. Aside from the IL-6cD3 and PCTD3 levels, all other variables were significantly different between the two groups.

**Table 2:** Biomarkers in blood of the study groups

Blood biomarkers	Survivor group (*n* = 314)	Nor-survivor group(*n* = 53)	*P* value
IL-6D1 (pg/ml)	85.70 (2.10 to 5000.00)	242.80 (16.45 to 5000.00)	0.002*
IL-6D3 (pg/ml)	39.59 (0.32 to 622.60)	185.00 (2.32 to 5000.00)	0.000*
IL-6cD3 (%)	47.06 (− 2786.85 to 99.62)	50.00 (− 546.38 to 39.71)	0.384
LACD1 (mmol/l)	2.12 (0.80 to 27.11)	2.20 (1.00 to 31.1)	0.036*
LACD3 (mmol/l)	1.70 (0.19 to 62.23)	2.40 (0.89 to 24.43)	0.000*
LACcD3 (%)	21.58 (− 2380 to 91.32)	− 13.33 (− 280 to 90.45)	0.000*
PCTD1 (ng/ml)	19.86 (0.05–200.00)	4.62 (0.06 to 130.17)	0.002*
PCTD3 (ng/ml)	6.60 (0.04–200.00)	7.03 (0.08 to 200.00)	0.130
PCTcD3 (%)	58.50 (− 11,418.75 to 99.43)	–15.07 (–2128.36 to 75.71)	0.000*

Data are presented as medians (range)

*PCT* procalcitonin, *LAC* lactate, *IL-6* Interleukin 6

^*^Indicates a significant value, *P* < 0.05

### Comparison of IL-6, LAC, and PCT levels, and clearance between the training and validation cohorts

Table 3 shows a comparison of the variables between the training and validation cohorts. There were no significant differences for all variables between the two groups.

**Table 3:** Biomarkers in blood of the training and validation cohort

Blood biomarkers	Training cohort (*n* = 257)	Validation cohort (*n* = 110)	*P* value
IL-6D1 (median [IQR])	102.30 [37.22, 315.30]	108.75 [46.71, 249.62]	0.766
IL-6D3 (median [IQR])	40.28 [18.99, 103.90]	52.61 [21.68, 109.97]	0.248
IL-6cD3 (median [IQR])	49.19 [− 3.94, 84.95]	45.46 [− 19.38, 83.35]	0.286
LACD1 (median [IQR])	2.13 [1.60, 2.80]	2.17 [1.60, 3.13]	0.631
LACD3 (median [IQR])	1.70 [1.30, 2.24]	1.74 [1.38, 2.50]	0.413
LACcD3 (median [IQR])	19.50 [− 7.95, 39.62]	20.26 [− 9.02, 37.43]	0.905
PCTD1 (median [IQR])	15.15 [2.35, 53.21]	19.06 [2.41, 83.03]	0.317
PCTD3 (median [IQR])	6.95 [1.30, 26.95]	6.94 [1.43, 35.05]	0.577
PCTcD3 (median [IQR])	52.77 [14.29, 73.19]	57.94 [25.00, 73.09]	0.529

*IL-6* interleukin 6, *LAC* lactate; *PCT* procalcitonin

### Logistic regression analysis for the training cohort

Table 4 shows the results of the logistic regression analysis. IL-6D3 (*OR* = 1.007; 95% *CI*: 1.003–1.010; *P* = 0.000), LACcD3 (*OR* = 0.993; 95% *CI*: 0.986–1.000; *P* = 0.042) and PCTD1 (*OR* = 0.957; 95% *CI*: 0.933–0.982; *P* = 0.001) are independent risk factors for 28-day sepsis mortality. Based on the AIC (164.335), PCTD1, PCTD3, IL-6D1, IL-6D3, and LACcD3 were used to develop the model.

**Table 4:** The logistic regression analysis of IL-6, LAC and PCT levels, and clearance for predicting 28-day mortality in 257 patients with sepsis or septic shock for training cohort

	Univariate analysis		Multivariate analysis	
Variables	*OR* (95% *CI*)	*P* value	*OR* (95% *CI*)	*P* value
IL-6D1 (pg/ml)	1.000 (1.000, 1.001)	0.004	1.000 (1.000, 1.001)	0.128
IL-6D3 (pg/ml)	1.006 (1.003, 1.009)	0.000	1.007 (1.003, 1.010)	0.000
IL-6cD3 (%)	0.999 (0.998, 1.001)	0.409		
LACD1 (mmol/l)	1.197 (1.040, 1.377)	0.012		
LACD3 (mmol/l)	1.077 (0.985, 1.178)	0.102		
LACcD3 (%)	0.991 (0.985, 0.996)	0.002	0.993 (0.986, 1.000)	0.042
PCTD1 (ng/ml)	0.988 (0.979, 0.997)	0.012	0.957 (0.933, 0.982)	0.001
PCTD3 (ng/ml)	1.010 (1.002, 1.018)	0.015	1.022 (0.996, 1.048)	0.093
PCTcD3 (%)	1.000 (1.000, 1.000)	0.209		

*OR* odds ratio, *CI* confidence interval, *IL-6* interleukin 6, *LAC* lactate, *PCT* procalcitonin

### Performance of the prediction model shown by ROC, calibration, and DCA curves

**Fig. 2:**
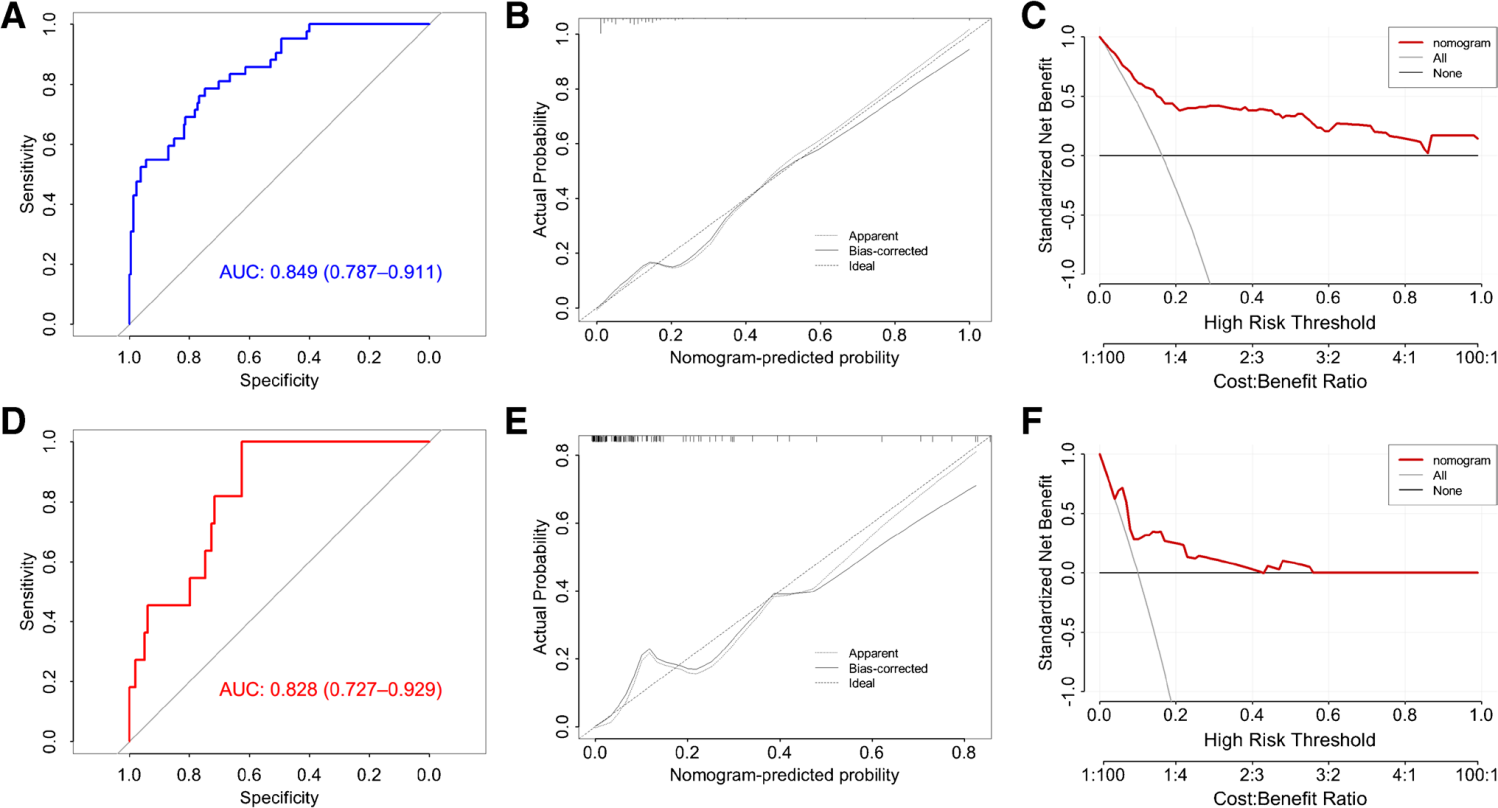
Performance of the prediction model. **A**, **B**, and **C** show the ROC, calibration, and DCA curves of the training cohort, and **D**, **E**, and **F** show those of the validation cohort, respectively. The ROC results show that the models in the training cohort (0.849, 95% *CI*: 0.0.787–0.911) and validation cohort (0.828, 95% *CI*: 0.0.727–0.929), both have good prediction accuracy. The calibration plot shows that the predicted values are consistent with the actual values. The DCA curve shows that there are positive benefits in the training cohort and the validation cohort. The training cohort has positive benefits in all thresholds, while the validation cohort has positive benefits below the threshold of 0.55. ROC, receiver operating characteristic curves; DCA, decision curve analysis

**Fig. 3:**
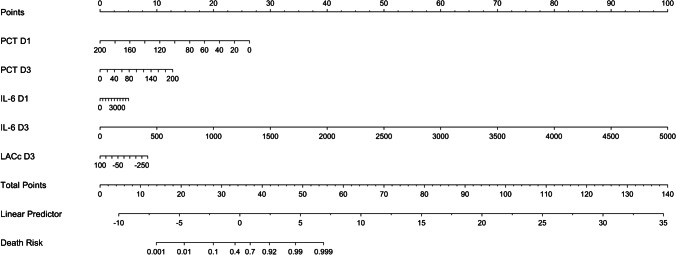
Nomogram to assess the risk of death in septic patients. To use the nomogram, first, a line is drawn from each indicator value to the points line to obtain the score. The points for all indicators are then added. Lastly, a line from the total points line to the lowest line of the nomogram is drawn to determine the risk of death

Fig. 2A, B, and C show the ROC, calibration, and DCA curves of the training cohort, while Fig. 2D, E, and F are those of the validation cohort. The ROC results revealed that the models in the training cohort (0.849, 95% *CI*: 0.0.787–0.911) and validation cohort (0.828, 95% *CI*: 0.0.727–0.929), both had good prediction accuracy. The calibration plot showed that the predicted values were consistent with the actual values. The DCA curve showed that there were positive benefits in the training and validation cohorts. The training cohort had positive benefits for all thresholds, whereas the validation cohort was positive below the threshold of 0.55. Figure 3 shows that the risk nomogram verified the predictive value of the model

## Discussion

This retrospective single-center study demonstrated that combining the levels and clearance of IL-6, LAC, and PCT was valuable for predicting 28-day sepsis mortality. Specifically, the present study showed that combining IL-6D1, IL-6D3, PCTD1, PCTD3, and LACcD3 can provide a simple prediction model for 28-day sepsis mortality with good performance and good operability.

As a major pro-inflammatory cytokine, IL-6 levels increase in patients with infection, as well as with trauma, surgery, and neoplastic infarction [13, 30]. Several previous studies have demonstrated that IL-6 levels are correlated with sepsis mortality [17, 31–33]. Research suggests that a combination of initial and follow-up IL-6 levels could provide an additional prognostic value for sepsis mortality [16]. Another study reported that the prognostic value of IL-6 levels for 28-day sepsis mortality increased over time, up to 7 days [18]. In our study, IL-6D1 and IL-6D3 levels were significantly higher in non-survivors than in survivors. Although IL-6D3 was the only independent risk factor for 28-day sepsis mortality, both IL-6D1 and IL-6D3 were beneficial in the prediction model. However, IL-6 clearance was not a risk factor for sepsis in our study; thus, it was not used in developing the prediction model. There are very few studies on IL-6 clearance. A previous study found that continuous hemofiltration increases IL-6 plasma clearance but cannot change its plasma concentration effectively in systemic inflammatory response syndrome (SIRS) [34]. This suggests that controlling the production of IL-6, instead of IL-6 clearance, may be the key to treating infections.

As a marker of tissue hypoxia, LAC has been widely used as a prognostic marker for sepsis. According to the Sepsis-3 criteria, LAC concentration is a critical marker for the diagnosis of septic shock. Patients with septic shock often have sustained or increased LAC levels, which may be due to either increased LAC production or impaired LAC clearance [35]. In addition, lactic acidosis is common in sepsis and causes a higher mortality rate in septic patients [27, 36]. As LAC levels indicate a balance between elimination and production, continuous monitoring may be more effective than a single measurement. During resuscitation, organ function often improves along with high LAC clearance, which is beneficial for decreasing the mortality risk. Our study showed that LACcD3 was a valuable predictor of 28-day mortality in septic patients. Although several previous studies have shown that increased LAC levels are a valuable prognostic marker of high mortality in sepsis [16, 17, 37, 38], another study showed that critically ill patients with sepsis had normal serum LAC levels [39]. Therefore, it is better to collect serial LAC measurements and combine LAC levels with other markers to evaluate sepsis prognosis more accurately.

PCT was first used as a biomarker of infection in 1993 [40]. Some studies have found that serum PCT concentrations are correlated with the severity of sepsis [24]. Currently, it is one of the most accepted biomarkers for the prognostic prediction of sepsis-related mortality. Previous research has indicated that the change range of PCT may be different from its baseline level; therefore, the concept of clearance was used [41]. In previous studies, serum PCT measured on days 1, 3, and 5 of ICU stay was not predictive of mortality [26], but PCT clearance significantly decreased in non-survivors, compared to survivors within 48 h. Conversely, in the present study, PCT clearance was not an independent risk factor for predicting 28-day sepsis mortality. One reason for this may be that we did not focus on finding the independent risk factors but looked for one of the best models in logistic regression analysis; thus, we used stepwise regression to construct the model. The step-by-step method combines the advantages of the forward and backward methods. After each new independent variable is introduced, the substituted independent variable must be recalculated to check whether it has the value of remaining in the equation. Based on this, the introduction and elimination of independent variables are alternately carried out until no new variable can be introduced or eliminated. Another reason may be the sample size, as a previous study only enrolled 48 patients, including eight non-survivors. Since our sample size is not sufficiently large, further research is needed to confirm these results.

This study had several advantages. First, the enrolled patients were diagnosed based on the latest Sepsis-3 criteria. Second, we serially measured the concentrations of three potential biomarkers of sepsis, including IL-6, LAC and PCT. Third, we calculated the clearance of IL-6, LAC, and PCT at different time points. Fourth, the prognostic values of both the levels and clearance of these markers were demonstrated. To the best of our knowledge, this study is the first to evaluate the combined predictive performance of IL-6, LAC, and PCT levels, and clearance. Owing to the small amount of data, the main purpose of the present study was to preliminarily evaluate the predictive value of continuous monitoring of these indicators for sepsis. In future studies, we will collect more cases to complete a more rigorous prediction model and simultaneously verify the model at the same time.

### Study limitation

This study has some limitations. First, it was performed at a single center. Multicenter-based research with a larger quantity of data will still be necessary to confirm the results. Second, we excluded patients hospitalized for less than 24 h, and most of these patients died. For these critically ill patients, biomarkers with early diagnostic and prognostic values are urgently needed. Third, the results of this study may not adapt universally to other septic patients in ER because of our exclusion criteria. Such as cancer patients were excluded in our study, so further research is necessary to verify whether this result is applicable to tumor patients with sepsis.

## Conclusions

The combination of IL-6D1, IL-6D3, PCTD1, PCTD3, and LACcD3 may be used to build a prediction model for 28-day sepsis mortality. These results still need to be verified in a larger sample size through multicenter-based research.

## Data Availability

The data for this study may be available by contacting the corresponding author upon reasonable request.

## References

[1] Rudd KE, Johnson SC, Agesa KM (2020). Global, regional, and national sepsis incidence and mortality, 1990–2017: analysis for the Global Burden of Disease study. The Lancet.

[2] Singer M, Deutschman CS, Seymour CW (2016). The third international consensus definitions for sepsis and septic shock (Sepsis-3). JAMA.

[3] Zhou X, Su LX, Zhang JH (2019). Rules of anti-infection therapy for sepsis and septic shock. Chin Med J (Engl).

[4] Evans IVR, Phillips GS, Alpern ER (2018). Association between the New York sepsis care mandate and in-hospital mortality for pediatric sepsis. JAMA.

[5] Liu L, Han Z, An F (2021). Aptamer-based biosensors for the diagnosis of sepsis. J Nanobiotechnol.

[6] Seymour CW, Gesten F, Prescott HC (2017). Time to treatment and mortality during mandated emergency care for sepsis. N Engl J Med.

[7] Czajka S, Ziebinska K, Marczenko K (2020). Validation of APACHE II, APACHE III and SAPS II scores in in-hospital and one year mortality prediction in a mixed intensive care unit in Poland: a cohort study. BMC Anesthesiol.

[8] Haas LEM, Termorshuizen F, de Lange DW (2020). Performance of the quick SOFA in very old ICU patients admitted with sepsis. Acta Anaesthesiol Scand.

[9] Hou N, Li M, He L (2020). Predicting 30-days mortality for MIMIC-III patients with Sepsis-3: a machine learning approach using XGboost. J Transl Med.

[10] Wang J, Sun Y, Teng S (2020). Prediction of sepsis mortality using metabolite biomarkers in the blood: a meta-analysis of death-related pathways and prospective validation. BMC Med.

[11] Collins GS, Reitsma JB, Altman DG (2015). Transparent reporting of a multivariable prediction model for individual prognosis or diagnosis (TRIPOD): the TRIPOD statement. J Clin Epidemiol.

[12] Li L, Chen L, Lin F (2021). Study of the expression of inflammatory factors IL-4, IL-6, IL-10, and IL-17 in liver failure complicated by coagulation dysfunction and sepsis. J Inflamm Res.

[13] Smok B, Domagalski K, Pawlowska M (2020). Diagnostic and prognostic value of IL-6 and sTREM-1 in SIRS and sepsis in children. Mediators Inflamm.

[14] Hung SK, Lan HM, Han ST et al (2020) Current Evidence and Limitation of Biomarkers for Detecting Sepsis and Systemic Infection. Biomed 8(11) 10.3390/biomedicines8110494 10.3390/biomedicines8110494 PMC7697922 33198109

[15] Rios-Toro JJ, Marquez-Coello M, Garcia-Alvarez JM (2017). Soluble membrane receptors, interleukin 6, procalcitonin and C reactive protein as prognostic markers in patients with severe sepsis and septic shock. PLoS One.

[16] Song J, Park DW, Moon S (2019). Diagnostic and prognostic value of interleukin-6, pentraxin 3, and procalcitonin levels among sepsis and septic shock patients: a prospective controlled study according to the Sepsis-3 definitions. BMC Infect Dis.

[17] Miguel-Bayarri V, Casanoves-Laparra EB, Pallas-Beneyto L (2012). Prognostic value of the biomarkers procalcitonin, interleukin-6 and C-reactive protein in severe sepsis. Med Intensiva.

[18] Takahashi W, Nakada TA, Yazaki M (2016). Interleukin-6 levels act as a diagnostic marker for infection and a prognostic marker in patients with organ dysfunction in intensive care units. Shock.

[19] Hui S, Ghergurovich JM, Morscher RJ (2017). Glucose feeds the TCA cycle via circulating lactate. Nature.

[20] Lee SG, Song J, Park DW (2021). Prognostic value of lactate levels and lactate clearance in sepsis and septic shock with initial hyperlactatemia: a retrospective cohort study according to the Sepsis-3 definitions. Med (Baltimore).

[21] Innocenti F, Meo F, Giacomelli I (2019). Prognostic value of serial lactate levels in septic patients with and without shock. Intern Emerg Med.

[22] Ryoo SM, Lee J, Lee YS (2018). Lactate level versus lactate clearance for predicting mortality in patients with septic shock defined by Sepsis-3. Crit Care Med.

[23] Evans L, Rhodes A, Alhazzani W (2021). Surviving sepsis campaign: international guidelines for management of sepsis and septic shock 2021. Intensive Care Med.

[24] Hamade B, Huang DT (2020). Procalcitonin: where are we now?. Crit Care Clin.

[25] Mat Nor MB, Md Ralib A (2014). Procalcitonin clearance for early prediction of survival in critically ill patients with severe sepsis. Crit Care Res Pract.

[26] Huang MY, Chen CY, Chien JH (2016). Serum procalcitonin and procalcitonin clearance as a prognostic biomarker in patients with severe sepsis and septic shock. Biomed Res Int.

[27] Lee SM, Kim SE, Kim EB (2015). Lactate clearance and vasopressor seem to be predictors for mortality in severe sepsis patients with lactic acidosis supplementing sodium bicarbonate: a retrospective analysis. PLoS One.

[28] Huang ZY, Huang Q, Wang LY (2020). Normal trajectory of interleukin-6 and C-reactive protein in the perioperative period of total knee arthroplasty under an enhanced recovery after surgery scenario. BMC Musculoskelet Disord.

[29] Nassar AP, Nassif BN, Santos D (2020). Procalcitonin clearance at 24, 48, 72, and 96 hours and mortality in patients with cancer and sepsis: a retrospective cohort study. J Intensive Care Med.

[30] Sikora JP, Chlebna-Sokol D, Krzyzanska-Oberbek A (2001). Proinflammatory cytokines (IL-6, IL-8), cytokine inhibitors (IL-6sR, sTNFRII) and anti-inflammatory cytokines (IL-10, IL-13) in the pathogenesis of sepsis in newborns and infants. Arch Immunol Ther Exp (Warsz).

[31] Xie Y, Li B, Lin Y (2021). Combining blood-based biomarkers to predict mortality of sepsis at arrival at the Emergency Department. Med Sci Monit.

[32] Tschaikowsky K, Hedwig-Geissing M, Braun GG (2011). Predictive value of procalcitonin, interleukin-6, and C-reactive protein for survival in postoperative patients with severe sepsis. J Crit Care.

[33] Hack CE, De Groot ER, Felt-Bersma RJ (1989). Increased plasma levels of interleukin-6 in sepsis. Blood.

[34] Sander A, Armbruster W, Sander B (1997). Hemofiltration increases IL-6 clearance in early systemic inflammatory response syndrome but does not alter IL-6 and TNF alpha plasma concentrations. Intensive Care Med.

[35] Kraut JA, Madias NE (2014). Lactic acidosis. N Engl J Med.

[36] Nguyen HB, Rivers EP, Knoblich BP (2004). Early lactate clearance is associated with improved outcome in severe sepsis and septic shock. Crit Care Med.

[37] Baysan M, Baroni GD, van Boekel AM (2020). The added value of lactate and lactate clearance in prediction of in-hospital mortality in critically ill patients with sepsis. Crit Care Explor.

[38] Sugimoto M, Takayama W, Murata K (2021). The impact of lactate clearance on outcomes according to infection sites in patients with sepsis: a retrospective observational study. Sci Rep.

[39] Sauer CM, Gomez J, Botella MR (2021). Understanding critically ill sepsis patients with normal serum lactate levels: results from U.S. and European ICU cohorts. Sci Rep.

[40] Assicot M, Gendrel D, Carsin H (1993). High serum procalcitonin concentrations in patients with sepsis and infection. Lancet.

[41] Ruiz-Rodriguez JC, Caballero J, Ruiz-Sanmartin A (2012). Usefulness of procalcitonin clearance as a prognostic biomarker in septic shock. Prospect Pilot Study Med Intensiva.

